# Rice Cultivar Takanari Has Higher Photosynthetic Performance Under Fluctuating Light Than Koshihikari, Especially Under Limited Nitrogen Supply and Elevated CO_2_

**DOI:** 10.3389/fpls.2020.01308

**Published:** 2020-09-01

**Authors:** Satoshi Ohkubo, Yu Tanaka, Wataru Yamori, Shunsuke Adachi

**Affiliations:** ^1^ Institute of Global Innovation Research, Tokyo University of Agriculture and Technology, Fuchu, Japan; ^2^ Graduate School of Agriculture, Kyoto University, Kyoto, Japan; ^3^ Graduate School of Agricultural and Life Sciences, Institute for Sustainable Agro-Ecosystem Services, The University of Tokyo, Nishitokyo, Japan; ^4^ College of Agriculture, Ibaraki University, Inashiki, Japan

**Keywords:** CO_2_, electron transport, nitrogen, non-steady-state photosynthesis, rice, stomatal conductance

## Abstract

Plants in the field experience dynamic changes of sunlight rather than steady-state irradiation. Therefore, increasing the photosynthetic rate of an individual leaf under fluctuating light is essential for improving crop productivity. The high-yielding *indica* rice (*Oryza sativa* L.) cultivar Takanari is considered a potential donor of photosynthesis genes because of its higher steady-state photosynthesis at both atmospheric and elevated CO_2_ concentrations than those of several Japanese commercial cultivars, including Koshihikari. Photosynthetic induction after a sudden increase in light intensity is faster in Takanari than in Koshihikari, but whether the daily carbon gain of Takanari outperforms that of Koshihikari under fluctuating light in the field is unclear. Here we report that Takanari has higher non-steady-state photosynthesis, especially under low nitrogen (N) supply, than Koshihikari. In a pot experiment, Takanari had greater leaf carbon gain during the initial 10 min after a sudden increase in irradiation and higher daily CO_2_ assimilation under simulated natural fluctuating light, at both atmospheric (400 ppm) and elevated (800 ppm) CO_2_ concentrations. The electron transport rate during a day under field conditions with low N supply was also higher in Takanari than in Koshihikari. Although the advantages of Takanari were diminished under high N supply, photosynthetic N use efficiency was consistently higher in Takanari than in Koshihikari, under both low and high N supply. This study demonstrates that Takanari is a promising donor parent to use in breeding programs aimed at increasing CO_2_ assimilation in a wide range of environments, including future higher CO_2_ concentrations.

## Introduction

Most of the research programs conducted to improve photosynthetic performance of leaves through genetic engineering and conventional breeding have examined CO_2_ assimilation rate (*A*) at steady-state conditions in stable light environments (Yamori et al., 2016; Sage et al., 2017; Simkin et al., 2019). However, plants grown in the field often experience fluctuating sunlight during the day due to clouds, wind, and self-shading, and they rarely display steady-state photosynthesis (Pearcy, 1990; Pearcy et al., 1996; Leakey et al., 2004; Lawson et al., 2012; Slattery et al., 2018). In a soybean (*Glycine max* (L.) Merr.) canopy, for example, sunflecks contribute 40–90% of daily photosynthetic photon flux density (PPFD), with approximately one-third contributed by sunflecks shorter than 10 s (Pearcy, 1990). When the crop canopy is exposed to a sunfleck, leaf *A* gradually increases to reach a new steady-state level, which takes several seconds to minutes (Pearcy, 1990; Yamori, 2016; Tanaka et al., 2019). This process, which is termed photosynthetic induction, may reduce photosynthetic light use efficiency, and the photosystems may be damaged by excess sunlight (Yamori, 2016). The carbon loss due to photosynthetic induction was estimated to be at least 21.0% in wheat and 21.2% in soybean relative to steady-state light (Taylor and Long, 2017; Tanaka et al., 2019). Therefore, improving non-steady-state photosynthesis under fluctuating light is an essential challenge to increase crop productivity.

Natural genetic resources are potential donors for improvement of leaf photosynthesis (Flood et al., 2011; van Bezouw et al., 2019; Adachi et al., 2020). In rice (*Oryza sativa* L.), the steady-state *A* of the uppermost expanded leaves at reproductive stages varies from 11.9 to 32.1 µmol m^−2^ s^−1^ among 65 cultivars and landraces from the ‘world core collection’ harboring approximately 90% of worldwide allelic variation (Kojima et al., 2005; Kanemura et al., 2007). A wide range of genetic variation in steady-state *A* has also been observed among cultivars and breeding lines in rice (Jahn et al., 2011; Qu et al., 2017), wheat (*Triticum aestivum* L.; Carmo-Silva et al., 2017), soybean (Sakoda et al., 2016), and maize (*Zea mays* L.; Choquette et al., 2019). Quantitative trait locus analysis and genome-wide association studies were conducted to use these variations in crop breeding (Adachi et al., 2011; Adachi et al., 2019b; Wang et al., 2020). Very recently, Acevedo-Siaca et al. (2020) reported a difference of 109% in the total amount of CO_2_ fixed during the first 5 min of induction after a sudden increase in irradiance (CCF_5_) among 14 rice accessions. Wide differences of carbon gain during photosynthetic induction between cultivars have also been observed in wheat with percentage genetic difference of 80% in the time required for 95% induction (Salter et al., 2019), and soybean with percentage genetic difference of 580% in CCF_5_ (Soleh et al., 2016; Soleh et al., 2017). However, our understanding of genetic variation of non-steady-state photosynthesis is still limited relative to that of steady-state photosynthesis, which prohibits to develop breeding strategies for enhancing canopy photosynthetic capacity in the field conditions (Tanaka et al., 2019).

Koshihikari and Takanari have been considered model rice cultivars for the past decade owing to the contrasting properties of their photosynthesis (Taylaran et al., 2011). The *japonica* cultivar Koshihikari is the most widely grown cultivar in Japan owing to its high grain quality, despite its moderate photosynthetic capacity and grain yield (Adachi et al., 2020). The *indica* cultivar Takanari, developed for forage and processing rather than human consumption, has one of the highest grain yields among Japanese rice cultivars (Xu et al., 1997; Imbe et al., 2004). Takanari has one of the highest steady-state *A* values of the flag leaf among the 65 abovementioned cultivars (Kanemura et al., 2007). Its high *A* is largely explained by high leaf nitrogen (N) content due to high N accumulation, and high stomatal conductance (*g*_s_) due to large water uptake capacity (Taylaran et al., 2011; Muryono et al., 2017), and could be also associated with higher mesophyll conductance (*g*_m_) than that of Koshihikari (Chen et al., 2014). The high leaf photosynthetic capacity of Takanari, especially at the reproductive stage, increases crop growth rate and eventually yield (Xu et al., 1997; Takai et al., 2006; Taylaran et al., 2011). Koshihikari, Takanari, and their introgression lines have been widely used for genetic studies of photosynthesis (Takai et al., 2013; Adachi et al., 2019b) and other physiological traits (Takai et al., 2014; Ookawa et al., 2016; San et al., 2018). Takanari also has higher steady-state photosynthesis than Koshihikari at elevated CO_2_ in free-air CO_2_ enrichment (FACE) experiments (Chen et al., 2014; Ikawa et al., 2019). We have reported that Takanari has a greater photosynthetic induction response to a sudden increase in irradiance than Koshihikari, which could be explained by a combination of a faster response of electron transport rate, larger accumulation of metabolites in the Calvin cycle, and rapid elevation of *g*_s_ (Adachi et al., 2019a). Although Takanari has high steady-state *A* and fast photosynthetic induction, its photosynthesis must be evaluated under field conditions with fluctuating light for judgment of its potential value as a donor cultivar in rice breeding programs.

Nitrogen is the primary determinant of leaf photosynthesis because large amounts of leaf N are allocated to ribulose-1,5-bisphosphate carboxylase/oxygenase (Rubisco) and other photosynthetic proteins (Makino et al., 2003). Leaf N content is closely correlated with steady-state *A* in rice (Cook and Evans, 1983; Hirasawa et al., 2010). It was recently reported that elevated leaf N content enhances the photosynthetic induction response in rice (Sun et al., 2016). High N supply also alleviates heat stress under fluctuating light in rice (Huang et al., 2017). Therefore, N may play an important role in the non-steady-state photosynthesis as well as steady-state photosynthesis. Elevated CO_2_ concentration facilitates photosynthetic induction under fluctuating light (Leakey et al., 2002; Kaiser et al., 2017), which can be explained by a decreased limitation of CO_2_ diffusion from air into chloroplasts, increased post-irradiance CO_2_ fixation, and decreased post-irradiance CO_2_ burst (Leakey et al., 2004). It is reported that a poplar species with low stomatal conductance showed lower photosynthetic induction than another poplar species with high stomatal conductance under atmospheric CO_2_, but the difference was disappeared under elevated CO_2_ due to the decreased limitation of stomata (Tomimatsu and Tang, 2012). To develop breeding strategies to enhance photosynthesis under elevated CO_2_, the genetic interactions of photosynthetic induction with N supply and CO_2_ concentration should be investigated.

Here, we evaluated the photosynthetic responses of Koshihikari and Takanari to a sudden increase in irradiance and the daily photosynthetic carbon gain under fluctuating light conditions mimicking a typical summer day, with different N supplies and at different CO_2_ concentrations. We also examined the dynamics of electron transport rate using a pulse-amplitude-modulation (PAM) chlorophyll fluorometer in the field. We conclude that Takanari is a promising genetic resource for improving non-steady-state photosynthesis, especially under limited N supply at high CO_2_ concentrations.

## Materials and Methods

### Plant Materials and Growth Conditions for Pot Experiments

Seeds of rice cultivars Takanari and Koshihikari were sown in nursery boxes filled with artificial soil, and the seedlings were grown until the 4th leaf stage in a greenhouse in 2018. The minimum and maximum temperatures inside the greenhouse were approximately 10 and 35°C, respectively. The seedlings were transplanted into 3-L pots (one per pot) with two different rates of N fertilization. For the low N supply (LN), the pots were filled with a 14:3:3 (v/v/v) mixture of sand, paddy soil (an alluvial clay loam), and upland soil (a diluvial volcanic ash) containing fertilizer (0.3 g of N as ammonium nitrate, and 0.5 g each of P as P_2_O_5_ and K as K_2_O per pot). For the high N supply (HN), the pots were filled with a 1:1 (v/v) mixture of paddy and upland soils containing fertilizer (0.5 g of N as ammonium nitrate and 0.5 g each of P as P_2_O_5_ and K as K_2_O per pot); additional fertilizer (0.5 g of N as ammonium nitrate per pot) was applied at 36 and 50 days after transplanting. The pots were placed in an experimental field of Tokyo University of Agriculture and Technology in Fuchu, Japan (35°41′N, 139°29′E), and the level of standing water was maintained at 2 to 4 cm above the soil. The minimum and maximum temperatures during the growth were approximately 10 and 32°C, and peak PPFD value on clear days was 1,200 µmol photons m^−2^ s^−1^.

### Measurements and Calculations of Photosynthetic Induction

The response of photosynthesis to a sudden increase in irradiance was measured with a LI-6400XT portable photosynthesis system (Li-Cor, Lincoln, NE, USA) using a leaf chamber fluorometer (LI-6400-40). Plants in their pots were transferred to a dark room in the evening before each measurement. Next morning, leaves were enclosed in the leaf chamber of the LI-6400XT and adapted to the background irradiance (50 µmol photons m^−2^ s^−1^) at either 400 or 800 ppm CO_2_ for 20 min at 28°C of leaf chamber temperature. Then irradiance was suddenly increased to 1,500 µmol photons m^−2^ s^−1^. Gas exchange parameters were automatically recorded every 10 s. The *A* and *g*_s_ values during photosynthetic induction were individually fitted to sigmoidal curves according to Kaiser et al. (2017). Cumulative CO_2_ fixation (CCF_10_) after the step increase in irradiance was calculated as the integrated sum of *A* over 10 min. After the measurement of photosynthetic induction, the responses to different levels of PPFD and CO_2_ were measured according to Yamori et al. (2020). Maximum rates of carboxylation (*V*_cmax_) and electron transport (*J*_max_) were estimated from *A*–*C*_i_ response curves as in Adachi et al. (2017), except that some parameters were changed to those in Bernacchi et al. (2001) at 30°C which is the average value of leaf temperature during *A*–*C*_i_ response curve measurements (*K*_c_ = 690.05 µmol m^−2^ s^−1^, *K*_o_ = 353.02 mmol m^−2^ s^−1^, Γ^*^ = 55.22 µmol mol^−1^, *R*_d_ = 1.37 µmol m^−2^ s^−1^). The biochemical and diffusional limitation during photosynthetic induction were analyzed according to Kaiser et al. (2017).

### Photosynthesis Under Simulated Field Light Condition

The diurnal changes in gas exchange rate were measured with the LI-6400XT and the LI-6400-40. To mimic a typical light environment in the canopy, PPFD values were recorded in the paddy field as in Adachi et al. (2019a). Typical daily light conditions over 12 h were replicated in the LI-6400XT chamber under the control of an auto-measuring program. Plants in their pots were transferred to a dark room in the evening before each measurement. The same leaf as was used for the photosynthetic induction measurement was also used for this measurement, but in a different part to avoid damage from the previous measurement. The leaf was enclosed in the leaf chamber at 05:55, kept in the dark for 3 min, and then irradiated at 70 µmol photons m^−2^ s^−1^ for 2 min. The auto-measuring program was initiated at 06:00. Gas exchange parameters were automatically recorded every 10 s and chlorophyll fluorescence parameters every 2 min for 12 h. Leaf chamber temperature was maintained at 28°C and CO_2_ concentration at either 400 or 800 ppm. The air flow rate into the chamber was set to 300 µmol s^−2^. The leaf-to-air vapor pressure difference was 1.1−1.3 kPa. The blue and red ratio of the actinic light by LED-irradiance light source was kept at 10 and 90%, respectively. The intensity of a modulated measuring beam was 0.01 µmol photons m^–2^ s^–1^ during measurements. The steady-state fluorescence (*F*_s_) and maximum fluorescence during a light-saturating pulse of ~8000 µmol photons m^–2^ s^–1^ (*F*_m_') *via* multiple turnover flash were measured. The electron transport rate (ETR) through photosystem II (PSII) was calculated as:

(1) ETR=0.5×0.84×(Fm'−Fs)/Fm'×PPFD

where 0.5 is the fraction of absorbed light reaching PSII and 0.84 is the leaf absorptance (Kumagai et al., 2010; Dallagnol et al., 2015; Yamori et al., 2020). The value of nonphotochemical quenching (NPQ) was also calculated following to Yamori et al. (2011) and Shimadzu et al. (2019). The total daily carbon gain (integrated *A*) was calculated as the integrated sum of *A* values over the 12-h experiment.

### Quantification of Nitrogen, Rubisco, and Chlorophyll Contents

Immediately after gas exchange measurement for 12 h, two 30-mm-long segments were excised from the center of each leaf used for the gas exchange measurements. One segment was quickly frozen in liquid nitrogen and stored at −80°C, and the other was dried at 80°C for 24 h. The dried sample was used for quantification of nitrogen content with a CN analyzer (MT700 Mark II, Yanako, Kyoto, Japan). The frozen sample was used for quantification of Rubisco and chlorophyll contents according to Yamori et al. (2012) with slight modiﬁcations. In brief, the leaf was ground in liquid nitrogen with a mortar and pestle, and homogenized in 800 µl of extraction buffer (50 mM HEPES, pH 7.8, 10 mM MgCl_2_, 1 mM EDTA·2Na, 0.1% [v/v] Triton X-100, 1.6% [w/v] polyvinyl pyrrolidone, and 5 mM (±)-dithiothreitol) and 24 µl of 25× cOmplete Protease Inhibitor Cocktail (Roche Diagnostics, Basel, Switzerland). After thorough mixing, a 100-µl aliquot was used for quantiﬁcation of chlorophyll content, and the rest of the homogenate was used for quantiﬁcation of Rubisco content. Chlorophylls were extracted with 80% (v/v) acetone and the concentrations of chlorophylls *a* and *b* were determined as in Porra et al. (1989). For Rubisco quantification, the homogenate was centrifuged (19,000× *g*, 4°C, 1 min) and 100-µl of supernatant was mixed with an equal volume of sample buffer (125 mM Tris·HCl, pH 6.8, 4% sodium dodecyl sulfate, 15% glycerol, and 0.02% bromophenol blue). The sample was heated at 95°C for 6 min and centrifuged (25,000× *g*, room temperature, 2 min). Proteins in the supernatant were separated by polyacrylamide gel electrophoresis (4.5% stacking gel and 12.5% resolving gel). The gel was stained with GelCode Blue Stain Reagent (Thermo Scientific, Waltham, MA, USA) and scanned with an optical scanner. The amount of Rubisco large subunit was determined in ImageJ software (National Institutes of Health, https://imagej.nih.gov/ij/) using bovine serum albumin as standard.

### Chlorophyll Fluorescence Analysis Under Field Conditions

Seeds of Takanari and Koshihikari were sown in nursery boxes filled with artificial soil, and the seedlings were grown until the 4th leaf stage in a greenhouse in 2019. Plants were transplanted into a paddy field of Tokyo University of Agriculture and Technology (35°39′N, 139°28′E) on 22 May with the chemical fertilizers of 30 kg N, 60 kg P, and 60 kg K ha^−1^ as a basal dressing. The plant density was 22.2 plants m^−2^ (at a spacing of 30 cm × 15 cm). The experimental plots (each 4 m × 4.5 m) was arranged in a complete randomized block design with three replicates. To avoid border effects, a single plant near the center of each plot was selected for the measurements. Diurnal changes in chlorophyll fluorescence parameters were measured with six measuring heads attached to a Monitoring-PAM fluorometer (Waltz, Effeltrich, Germany) as described in Ikeuchi et al. (2014) and Ishida et al. (2014). Three measuring heads were applied for Koshihikari leaves and another three for Takanari leaves. The leaf clips of the fluorometer were fixed on the south-facing upright leaves. The fluorescence parameters and PPFD of sunlight were recorded every 3 min from 04:48 to 18:48 on 14 August. ETR was calculated as described above.

### Statistical Analysis

All statistical analyses were performed in the R environment (R Core Team, 2018). The effects of genotype and N application on leaf N, Rubisco and chlorophyll contents, and parameters of steady-state photosynthesis were analyzed using two-way analysis of variance (ANOVA). The significance of the differences among four experimental groups (genotypes × N treatments) was tested with Tukey–Kramer’s test using the *multcomp* R package. The differences in CCF_10_ and integrated *A* among eight experimental groups (genotypes × N treatments × CO_2_ partial pressures) were also tested with Tukey–Kramer’s test.

## Results

### Steady-State Photosynthesis Under Different N Levels

The contents of leaf N, Rubisco, and chlorophyll, and steady-state photosynthesis values of plants grown in pots at different N levels are shown in **Table 1**. Under LN, leaf N and Rubisco contents were similar in Koshihikari and Takanari, but chlorophyll content was slightly higher in Takanari. Takanari had higher steady-state *A*_400_ by 29.8% and *A*_800_ by 32.0% than Koshihikari, and also had higher photosynthetic parameters including *g*_s400_, *g*_s800_, ETR_400_, ETR_800_, *V*_cmax_, and *J*_max_ than Koshihikari. Thus, Takanari had higher photosynthetic capacity than Koshihikari under LN regardless of CO_2_ concentrations. Under HN, leaf N content was lower, but Rubisco and chlorophyll contents were slightly higher in Takanari than in Koshihikari. Takanari had significantly lower values of ETR_400_ and *J*_max_ and tended to have lower *A*_400_, *A*_800_, ETR_800_, and *V*_cmax_ than Koshihikari. These results indicate that an increase in N supply led to a higher N leaf accumulation in Koshihikari than in Takanari, resulting in higher photosynthetic capacity of Koshihikari. Takanari had higher *A* and ETR values at different values of *C*_i_ under LN, and lower *A* and ETR values under HN compared with Koshihikari (**Supplementary Figure S1**). Similar results were obtained for *A*_400_ and *A*_800_ responses to PPFD (**Supplementary Figure S2**).

**Table 1:** Leaf contents of N, Rubisco, and chlorophyll, and parameters of steady-state photosynthesis.

	Koshihikari		Takanari		ANOVA		
	LN	HN	LN	HN	Nitrogen(N)	Cultivar(C)	N × C
N (mmol m^−2^)	71 ± 1^a^	136 ± 2^c^	71 ± 2^a^	110 ± 2^b^	***	***	***
Rubisco (µmol m^−2^)	2.13 ± 0.10^a^	4.37 ± 0.13^b^	2.10 ± 0.22^a^	4.79 ± 0.13^b^	***	N.S.	N.S.
Chlorophyll (µmol m^−2^)	85 ± 3^a^	221 ± 10^b^	106 ± 5^a^	244 ± 5^c^	***	**	N.S.
*A*_400_ (µmol m^−2^ s^−1^)	14.4 ± 0.5^a^	26.3 ± 1.9^c^	18.7 ± 0.7^ab^	23.2 ± 1.6^bc^	***	N.S.	*
ETR_400_ (µmol m^−2^ s^−1^)	156 ± 3^a^	235 ± 7^c^	177 ± 7^a^	202 ± 3^b^	***	N.S.	***
*g*_s400_ (mol m^−2^ s^−1^)	0.27 ± 0.02^a^	0.48 ± 0.07^b^	0.46 ± 0.03^b^	0.53 ± 0.04^b^	**	*	N.S.
*C*_i_/*C*_a 400_	0.74 ± 0.01^ab^	0.71 ± 0.01^a^	0.79 ± 0.01^c^	0.76 ± 0.01^bc^	**	***	N.S.
*A*_800_ (µmol m^−2^ s^−1^)	23.1 ± 0.6^a^	37.8 ± 1.4^c^	30.5 ± 0.9^b^	34.6 ± 0.7^c^	***	*	***
ETR_800_ (µmol m^−2^ s^−1^)	162 ± 12^a^	235 ± 2^c^	193 ± 3^b^	207 ± 6^bc^	***	N.S.	**
*g*_s800_ (mol m^−2^ s^−1^)	0.22 ± 0.01^a^	0.36 ± 0.02^b^	0.40 ± 0.02^b^	0.41 ± 0.03^b^	**	***	**
*C*_i_/*C*_a 800_	0.75 ± 0.01^a^	0.73 ± 0.02^a^	0.80 ± 0.01^b^	0.77 ± 0.01^ab^	*	**	N.S.
*V*_cmax_ (µmol m^−2^ s^−1^)	85 ± 2^a^	184 ± 6^c^	120 ± 4^b^	169 ± 7^c^	***	*	***
*J*_max_ (µmol m^−2^ s^−1^)	125 ± 2^a^	202 ± 6^d^	157 ± 4^b^	177 ± 5^c^	***	N.S.	***
*J*_max_/*V*_cmax_	1.47 ± 0.05^c^	1.10 ± 0.02^a^	1.31 ± 0.04^b^	1.05 ± 0.02^a^	***	*	N.S.

CO_2_ concentrations (400 or 800 ppm) used to measure steady-state photosynthetic parameters are indicated by subscript numbers. ***P < 0.001; **P < 0.01; *P < 0.05; N.S., not significant at 0.05 probability level. Values are means ± SE (n = 8). Values followed by the same letter do not differ significantly among groups at P < 0.05 by Tukey–Kramer multiple comparison test.

At the same leaf N content, *A*_400_ and *A*_800_ (**Supplementary Figure S3A**), *g*_s_ (**Supplementary Figure S3B**), *V*_cmax_ (**Supplementary Figure S3C**), and *J*_max_ (**Supplementary Figure S3D**) were higher in Takanari than in Koshihikari. These results indicate that Takanari had higher photosynthetic N use efficiency owing to the elevated photochemical capacity, enzymatic activity, and gas diffusion conductance per leaf N content.

### Initial Photosynthetic Carbon Gain After a Sudden Increase in Irradiation

**Figure 1:**
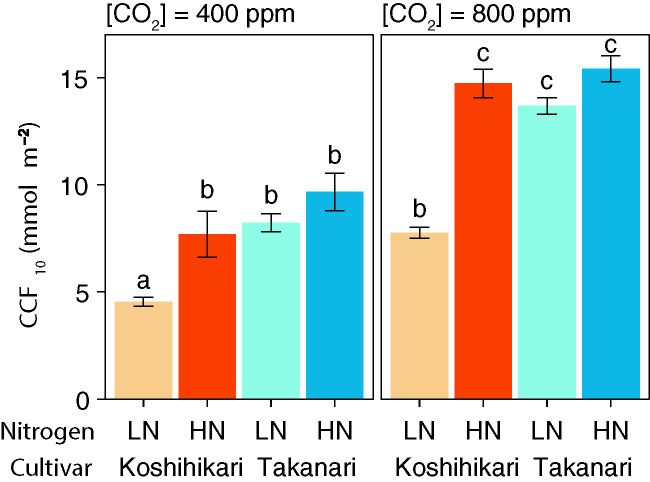
Cumulative CO_2_ fixation (CCF_10_) during the first 10 min of photosynthetic induction after transition from low to high irradiation at different CO_2_ concentrations. LN, low N supply; HN, high N supply. Values are means ± SE (*n* = 4). The same letter indicates no significant differences between groups at *P* < 0.05 by Tukey–Kramer multiple comparison test.

After a sudden increase in irradiation, the *A*_400_ and *A*_800_ values of Takanari rapidly increased and became higher than those of Koshihikari under LN, but were similar to those of Koshihikari under HN (**Supplementary Figures S4A, B**). The *g*_s_ value increased faster in Takanari than in Koshihikari under all experimental settings (**Supplementary Figures S4C, D**). At both CO_2_ concentrations, CCF_10_ was higher in Takanari under LN but not under HN (**Figure 1**). These results indicate that Takanari gains more carbon during photosynthetic induction under low N supply than does Koshihikari.

The biochemical limitation during photosynthetic induction was almost similar between the cultivars at any settings, except Koshihikari had higher biochemical limitation than Takanari at LN with 800 ppm CO_2_ (**Supplementary Figures S5A, B**). The diffusional limitation in Takanari at 400 ppm CO_2_ was remarkably lower than Koshihikari, while it was similar at 800 ppm CO_2_ (**Supplementary Figures S5C, D**). This result indicates that the elevated *g*_s_ in Takanari especially increased photosynthetic induction at the ambient CO_2_ condition. iWUE in Takanari during photosynthetic induction was consistently lower across the environments (**Supplementary Figures S4E, F**). This indicates that Takanari is inferior in terms of water use efficiency due to its excess transpiration.

### Photosynthetic Dynamics Under Simulated Fluctuating Light

**Figure 2:**
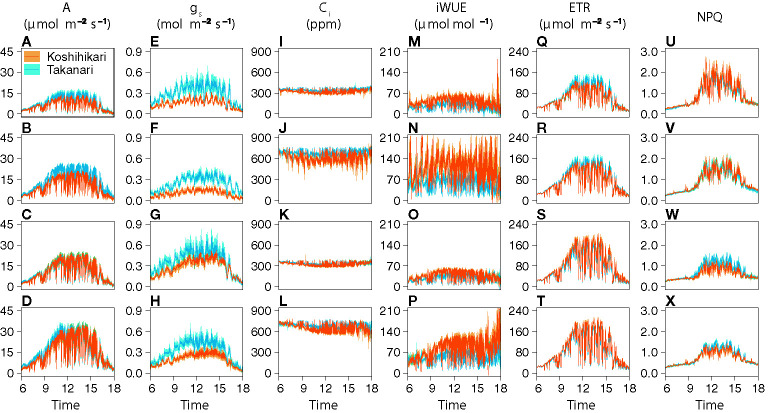
Diurnal patterns of photosynthesis under simulated light conditions mimicking a typical summer day. **(A–D)** CO_2_ assimilation rate (*A*), **(E–H)** stomatal conductance (*g*_s_), **(I–L)** internal CO_2_ concentration (*C*_i_), **(M–P)** intrinsic water use efficiency (iWUE), **(Q–T)** electron transport rate (ETR), and **(U–X)** nonphotochemical quenching (NPQ). The combinations of the levels of N fertilization (LN, low N supply; HN, high N supply) and CO_2_ concentrations (400 or 800 ppm) are shown on the left of the panels. Values are means ± SE (*n* = 4).

**Figure 3:**
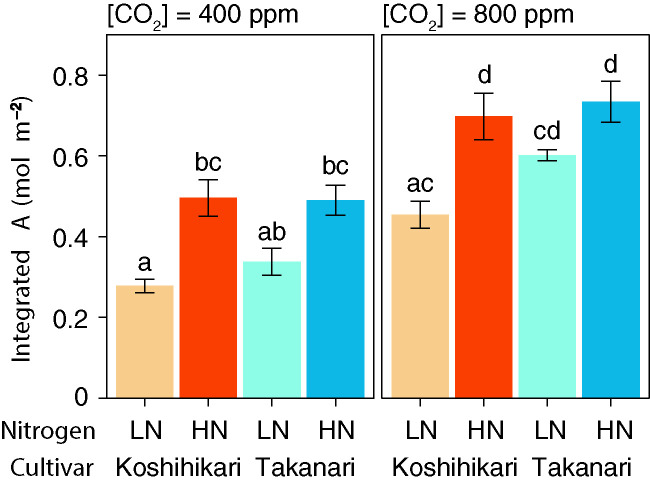
Total daily carbon gain (integrated *A*) under simulated light conditions at different CO_2_ concentrations. LN, low N supply; HN, high N supply. Data are integrated *A* values from **Figures 2A–D**. Values are means ± SE (*n* = 4). The same letter indicates no significant differences between groups at *P* < 0.05 by Tukey–Kramer multiple comparison test.

**Figure 4:**
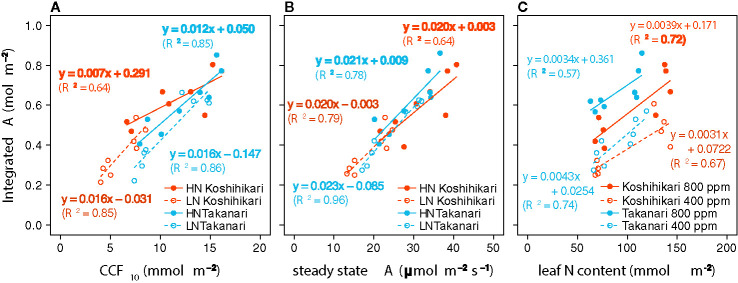
Relationships between total daily carbon gain (integrated *A*) and **(A)** cumulative CO_2_ fixation during the first 10 min of photosynthetic induction (CCF_10_), **(B)** steady-state CO_2_ assimilation rate (*A*), and **(C)** leaf nitrogen (N) content. Regression lines and equations are shown for individual cultivars and **(A, B)** N fertilization levels (LN, low N supply; HN, high N supply) or **(C)** CO_2_ concentrations. *R* ^2^ values are determination coefficients.

The auto-measuring program allowed gas exchange measurements under fluctuating light mimicking that in the field (**Figure 2**, **Supplementary Figure S6**). Under LN, Takanari had higher *A*, *g*_s_, and ETR than Koshihikari throughout the day at both CO_2_ concentrations (**Figures 2A, B, E, F, Q, R**, **Supplementary Table S1**). The NPQ values tended to be lower in Takanari, especially at the high light phase, however no significant difference in daily mean NPQ was found between Takanari and Koshihikari (**Figures 2U, V**, **Supplementary Table S1**). The superior photosynthesis under LN in Takanari was also evident from the plots of *A* and *g*_s_ *versus* PPFD (**Supplementary Figure S7**). Under HN, the *A* values were similar between the cultivars at both CO_2_ concentrations, whereas Takanari had higher *g*_s_ and lower ETR than Koshihikari (**Figures 2C, D, G, H, S, T**, **Supplementary Table S1**). NPQ values of Takanari tended to be higher than Koshihikari under HN, however no significant difference in daily mean NPQ was found (**Figures 2W, X**, **Supplementary Table S1**). The *C*_i_ value was significantly higher in Takanari than in Koshihikari under LN at 800 ppm CO_2_, and tended to be higher in the other treatments, indicating that CO_2_ diffusion from air (*i.e.*, CO_2_ supply) *versus* biochemical demand in Takanari was always higher than Koshihikari (**Figures 2I–L**, **Supplementary Table S1**). The value of iWUE of Takanari was lower than Koshihikari throughout a day at the most of settings, indicating that Takanari has lower water use efficiency at any environmental conditions (**Figures 2M–P**, **Supplementary Table S1**). The cumulative values of *A* (integrated over a day) tended to be higher under LN at both CO_2_ concentrations in Takanari than in Koshihikari, but were similar between the cultivars under HN (**Figure 3**). These values correlated with CCF_10_ irrespective of cultivar and treatment (**Figure 4A**), suggesting that a higher carbon gain during photosynthetic induction leads to a higher daily carbon gain under fluctuating light. Integrated *A* also correlated with steady-state *A* (**Figure 4B**), indicating that photosynthetic capacity is the primary determinant of the daily carbon gain. We also represented the correlation matrix of the observed parameters in **Supplementary Figure S8**.

At the same leaf N content and CO_2_ concentration, integrated *A* was higher in Takanari than in Koshihikari (**Figure 4C**). Hence, the photosynthetic N use efficiency is higher in Takanari than in Koshihikari not only under steady-state (**Supplementary Figure S3A**) but also non-steady-state conditions, regardless of CO_2_ concentration.

### Photosynthetic Dynamics Under Field Conditions

**Figure 5:**
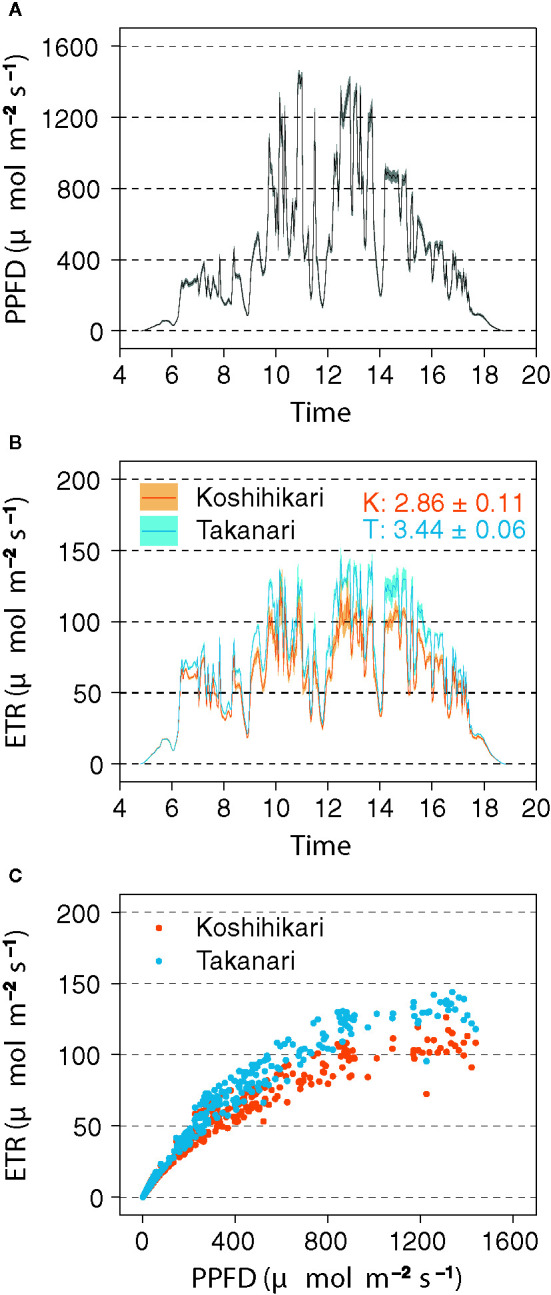
Electron transport rate (ETR) measured by a pulse-amplitude-modulation (PAM) chlorophyll fluorometer in the field. **(A)** Diurnal pattern of photosynthetic photon flux density (PPFD). Values are means ± SE (*n* = 6). **(B)** Diurnal patterns of ETR. Values are means ± SE (*n* = 3). Numbers within the panel are integrated ETR values (means ± SE, *n* = 3) of Koshihikari (K) and Takanari (T). **(C)** Relationship between ETR and PPFD. The data were recorded every 3 min from 04:48 to 18:48 on 14 August 2019.

In the field experiment, Takanari had higher ETR than Koshihikari, especially in the afternoon (**Figure 5B**), and a higher ETR in response to PPFD (**Figures 5A, C** **)**. The integrated value of ETR in Takanari (3.44 ± 0.06 mol m^−2^ day^−1^) was significantly higher in Koshihikari (2.86 ± 0.11 mol m^−2^ day^−1^) (*t*-test, *P* = 0.017). These results indicate that Takanari has high photosynthetic performance under natural field conditions.

## Discussion

Enhancing photosynthesis of field crops is essential for further increase of food production. Selection and characterization of potential donor cultivars with efficient photosynthesis would contribute to establishing breeding strategies for increasing yield (Flood et al., 2011; van Bezouw et al., 2019; Furbank et al., 2020). Here, we found that the high-yielding *indica* cultivar Takanari has higher *A* under fluctuating light than the Japanese commercial cultivar Koshihikari, especially under LN and elevated CO_2_ concentration.

Under LN and saturating irradiation, the steady-state *A*_400_ and *A*_800_ were higher in Takanari than in Koshihikari by approximately 30% (**Table 1**). This difference could be explained by the higher *g*_s_, *V*_cmax_, and *J*_max_ in Takanari, but not by the leaf N content (**Table 1**). Takanari had higher CCF_10_ at both CO_2_ concentrations (**Figure 1**), indicating a larger carbon gain during photosynthetic induction after a sudden increase in irradiance. At both CO_2_ concentrations, Takanari tended to have higher integrated *A* over a day under simulated fluctuating light conditions than Koshihikari (**Figure 3**). Takanari also showed higher ETR, a proxy for CO_2_ assimilation, in a field with relatively low N input (**Figure 5**). Thus, Takanari had higher photosynthesis under both steady-state and non-steady-state conditions at both CO_2_ concentrations tested. Higher steady-state photosynthesis in Takanari than in Koshihikari at a wide range of CO_2_ concentrations has been reported (Taylaran et al., 2011; Chen et al., 2014; Ikawa et al., 2018; Adachi et al., 2019b; Ikawa et al., 2019). The published data and our current study strongly suggest that Takanari is a promising donor cultivar for genes useful for enhancing photosynthesis under field conditions at current and future CO_2_ concentrations.

Excess light energy can induce photoinhibition, a photodamage of photosystems II and I, which is amplified by fluctuating light in natural field condition (Yamori, 2016). A leaf with lower photosynthetic capacity is subject to photoinhibition because the excess electrons accumulate in the photosystems and produce ROS within the thylakoid membranes, leading to degradation of photosynthetic proteins (Demmig-Adams et al., 1996). Therefore, photoinhibition in Koshihikari leaves under LN might be severer than Takanari, which could be anticipated by the lower *A* and the elevated thermal dissipation (*i.e.* NPQ) especially at the high light intensity during a day (**Figure 2**). Photoinhibition induced for several hours and days could decrease electron transport rate on the thylakoid membrane and eventually CO_2_ assimilation rate (Aro et al., 1993; Long et al., 1994). The difference in CO_2_ assimilation rate between Koshihikari and Takanari could be increased after several days of fluctuating light, which may enhance yield gap between the cultivars. The changes of photoinhibition for even longer period should be evaluated in future research.

Under HN, however, leaf photosynthesis differed less between the two cultivars than under LN, likely because of the higher leaf N accumulation in Koshihikari than in Takanari, resulting in similar *g*_s_ values and slightly higher *V*_cmax_ and *J*_max_ values in Koshihikari at both CO_2_ concentrations (**Table 1**). These properties decreased the difference in CCF_10_ and integrated *A* under HN at both CO_2_ concentrations (**Figures 1**–**3**). Although Takanari can take up larger amounts of N owing to its larger root system and greater hydraulic stream than those in Koshihikari, the difference in plant N uptake becomes smaller with increasing N supply (Adachi et al., 2011; Taylaran et al., 2011; Muryono et al., 2017; Hasegawa et al., 2019). Furthermore, the N allocation rate to the leaves is inherently lower in Takanari than in Koshihikari (Adachi et al., 2011; Muryono et al., 2017). These data suggest that Takanari accumulates a larger proportion of N in stems and roots than does Koshihikari under HN, resulting in similar leaf photosynthesis under both steady-state and non-steady-state conditions.

Steady-state *A* (**Supplementary Figure S3A**) and integrated *A* (**Figure 4C**) plotted as a function of leaf N were consistently higher in Takanari than in Koshihikari, regardless of N supply and CO_2_ concentration. This higher photosynthetic N use efficiency of Takanari was likely attributable to the higher *V*_cmax_, *J*_max_, and *g*_s_ per leaf N content (**Supplementary Figures S3B–D**). The higher *V*_cmax_ and *J*_max_ per leaf N content in Takanari probably indicate preferential allocation of leaf N to thylakoid membrane proteins and Calvin-cycle enzymes than to leaf structural components (Evans and Clarke, 2019). The higher *g*_s_ per leaf N content would be attributable to superior stomatal traits such as density and size of each stomatal pore (Ohsumi et al., 2007). The higher hydraulic conductance from roots to leaves, which increases leaf water potential, and higher cytokinin activity in the leaves also potentially associate with the higher *g*_s_ in Takanari (Taylaran et al., 2011; Adachi et al., 2019a). The above data show that photosynthetic N use efficiency is greater in Takanari than in Koshihikari, which would retain photosynthesis better with decreased N input (Hasegawa et al., 2019).

Doubling ambient CO_2_ concentration increased steady-state *A* by 44–60% in Koshihikari and by 49–63% in Takanari, regardless of N supply (**Table 1**). These values were similar to those for integrated *A* (40–61% and 49–76%, respectively; **Figure 3**). These results indicate that elevated CO_2_ affects the photosynthesis of Koshihikari and Takanari similarly regardless of light conditions. Elevated CO_2_ has additional effects on non-steady-state photosynthesis to those on steady-state photosynthesis by a decreased limitation of CO_2_ diffusion from air into chloroplasts, increased post-irradiance CO_2_ fixation, and decreased post-irradiance CO_2_ burst (Leakey et al., 2004; Tomimatsu and Tang, 2016). However, our results of integrated *A* at elevated CO_2_ did not show these effects. This might be caused by a lot of sunfleck events with high light leading to high CO_2_ fixation, masking the benefit during light fluctuation. The effects of elevated CO_2_ on total carbon gain are probably more important under light-limited environments such as the bottom of a crop canopy and the closed-forest understory, where the contribution of sunflecks to the daily carbon gain is greater than that in the top canopy (Leakey et al., 2002).

In general, elevated growth CO_2_ may decrease the contents of Rubisco and other soluble proteins due to the acclimation to high CO_2_ condition, reducing *V*_cmax_ and *J*_max_ (Ainsworth and Long, 2005). Here, we grew rice plants outdoors at the atmospheric CO_2_ concentration and hence we cannot identify the genetic difference in acclimatization to elevated CO_2_. In a FACE experiment (590 ppm CO_2_ on average), Takanari maintained higher *g*_s_, *V*_cmax_, and *J*_max_ than did Koshihikari, even though these values were slightly lower in both cultivars at the atmospheric CO_2_ concentration (390 ppm on average) (Chen et al., 2014). Therefore, we hypothesize that Takanari would have higher photosynthesis under fluctuating light and elevated growth CO_2_ than Koshihikari; this hypothesis must be examined in the future.

A close correlation between steady-state *A* and non-steady-state *A* was found regardless of cultivar and N fertilization level (**Figure 4B**), suggesting that genetic improvement of steady-state *A* may boost daily carbon gain under fluctuating light conditions. We identified several quantitative trait loci for steady-state *A* using several sets of introgression lines derived from Koshihikari and Takanari (Adachi et al., 2019b). Map-based cloning using the same lines identified a gene, *GREEN FOR PHOTOSYNTHESIS*, associated with the difference in steady-state *A* between Koshihikari and Takanari (Takai et al., 2013). We expect that these genetic factors will allow breeders to improve non-steady-state and steady-state *A*. Transgenic approaches are also expected to improve non-steady-state photosynthesis (Slattery et al., 2018). Kromdijk et al. (2016) showed that accelerating recovery from photoprotection by overproduction of three proteins in tobacco (*Nicotiana tabacum* L.) increased plant biomass under field conditions. Yamori et al. (2012) reported that the overexpression of Rubisco activase in rice accelerated photosynthetic induction, especially under heat stress. A mutant of *SLAC1*, encoding an anion channel of guard cells, has increased stomatal aperture and carbon gain during photosynthetic induction and under fluctuating light mimicking field conditions in rice (Yamori et al., 2020) and *Arabidopsis* (Kimura et al., 2020). Genetic modifications of thylakoid membrane proteins, Calvin-cycle enzymes, and proteins that control CO_2_ diffusion could independently promote non-steady-state photosynthesis. Combining natural alleles and transgenes could boost photosynthesis and plant growth under field conditions.

In conclusion, we found that Takanari has higher photosynthesis under fluctuating light, especially under limited N supply and elevated CO_2_, than Koshihikari. Photosynthetic N use efficiency was also higher in Takanari regardless of N supply and CO_2_ conditions. We propose that Takanari is a promising donor cultivar for genes useful in rice breeding aimed at increasing photosynthesis under current and future climates.

## Data Availability Statement

The original contributions presented in the study are included in the article/**Supplementary Material**; further inquiries can be directed to the corresponding authors.

## Author Contributions

YT, WY, and SA designed the experiments. SO and SA performed the experiments. SO, WY, and SA wrote the manuscript.

## Funding

This work was supported in part by the Japan Science and Technology Agency, CREST grant number JPMJCR15O2 (to SA), and by JSPS KAKENHI (grant numbers JP16H06552, JP18H02185 and JP18KK0170 to WY, and JP18K05585, JP19H02940, JP19H02939 and 19K05987 to SA).

## Conflict of Interest

The authors declare that the research was conducted in the absence of any commercial or financial relationships that could be construed as a potential conflict of interest.
