# Supplementary material for: Rice Cultivar Takanari Has Higher Photosynthetic Performance Under Fluctuating Light Than Koshihikari, Especially Under Limited Nitrogen Supply and Elevated CO2
Source: Front Plant Sci. 2020 Sep 1;11:1308. doi: 10.3389/fpls.2020.01308 (PMC7490297; doi:10.3389/fpls.2020.01308)
Supplement: Supplementary file 1 [file DataSheet_1.pdf]

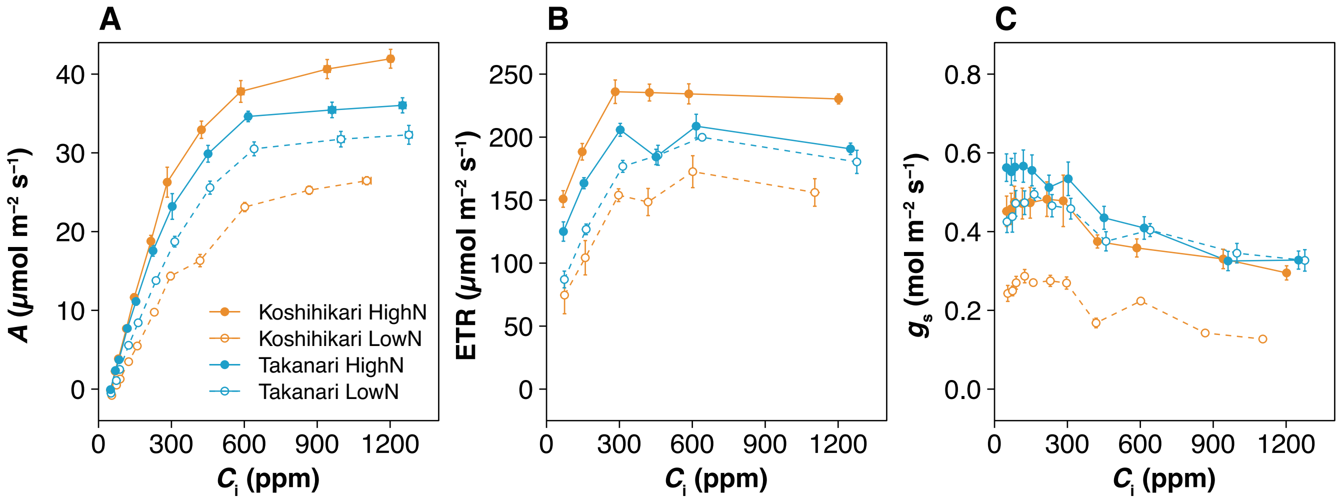


**Supplementary Figure S1** Responses of photosynthetic parameters to internal leaf $CO_2$ concentration ($C_i$). (**A**) $CO_2$ assimilation rate ($A$). (**B**) Electron transport rate through PSII (ETR). (**C**) stomatal conductance ($g_s$). Values are means ± SE ($n = 8$).

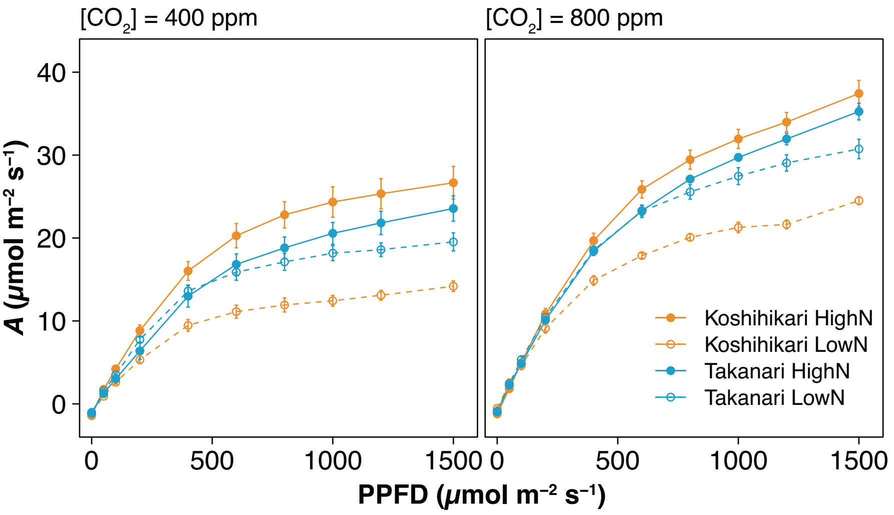


**Supplementary Figure S2** Responses of $CO_2$ assimilation rate ($A$) to photosynthetic photon flux density (PPFD) measured at different $CO_2$ concentrations. Values are means ± SE ($n = 4$).

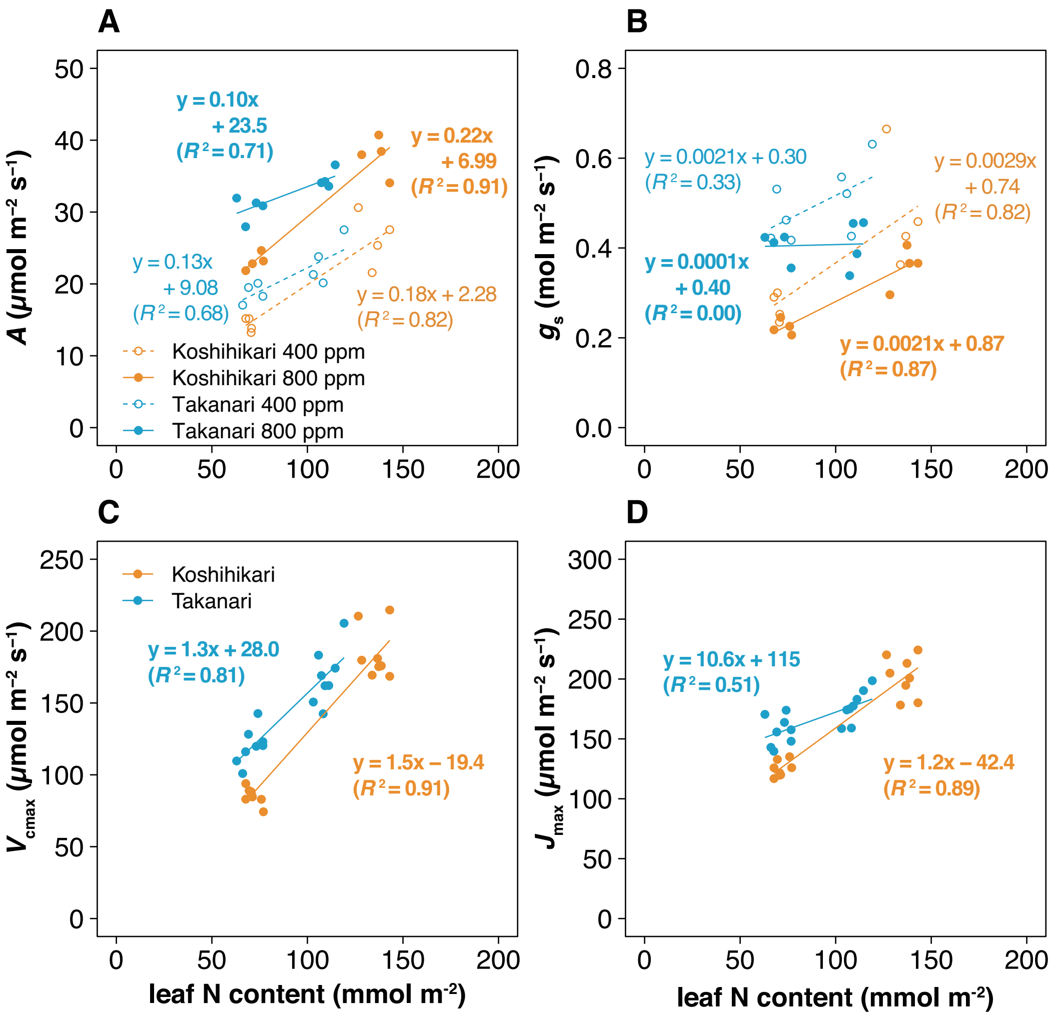


**Supplementary Figure S3** Relationships between gas exchange parameters and leaf N content. (**A**) $CO_2$ assimilation rate (*A*). (**B**) Stomatal conductance ($g_s$). (**C**) Maximum rate of carboxylation ($V_{cmax}$). (**D**) Maximum rate of electron transport ($J_{max}$). Regression lines and equations are shown. $R^2$ are determination coefficients.

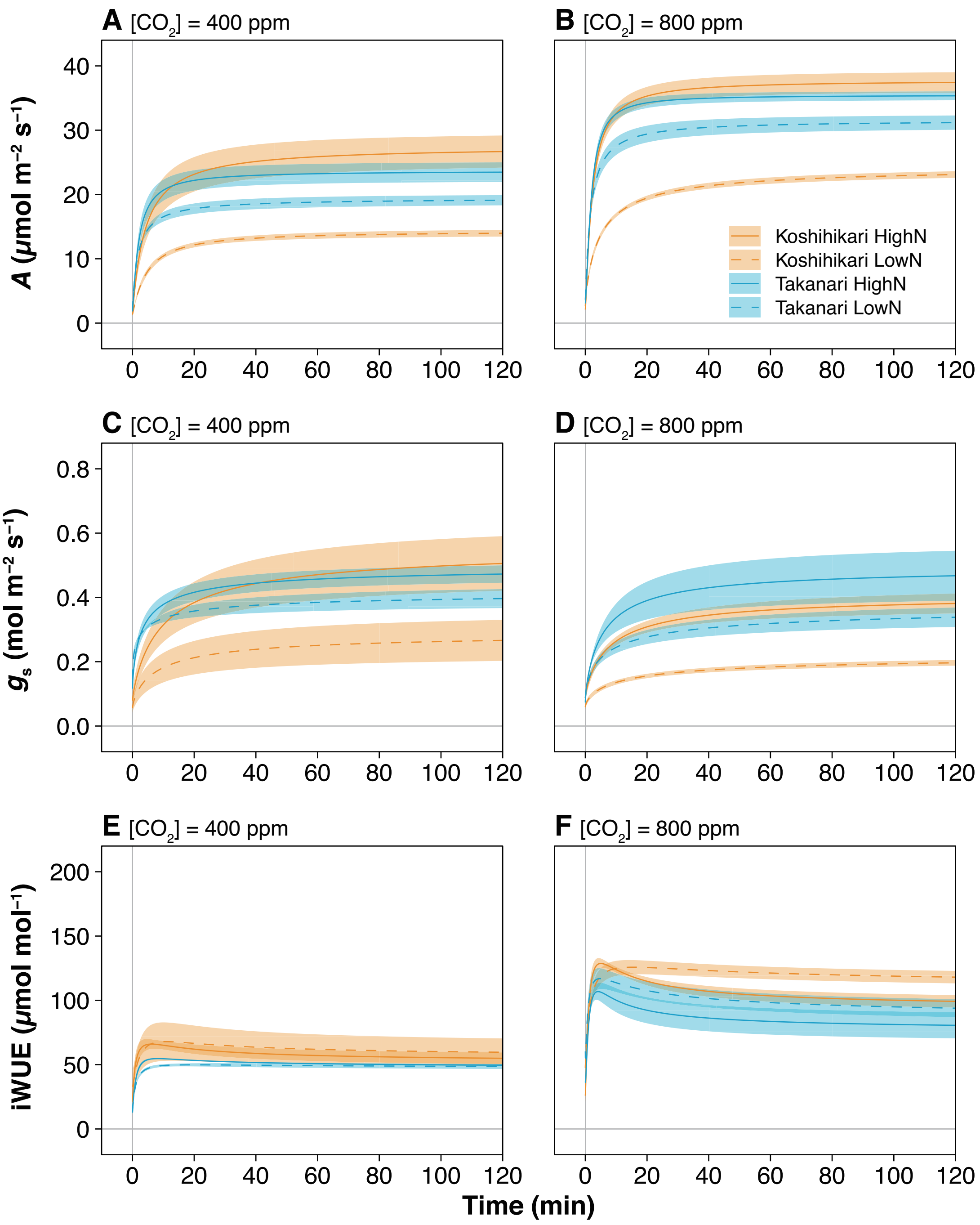


**Supplementary Figure S4** Dynamics of (**A, B**) $CO_2$ assimilation rate ($A$), (**C, D**) stomatal conductance ($g_s$) and (**E, F**) intrinsic water use efficiency (iWUE) after a sudden increase in irradiance measured at different $CO_2$ concentrations. Curves (means ± SE, $n = 4$) were fitted to the data using a sigmoidal function (Kaiser et al., 2017).

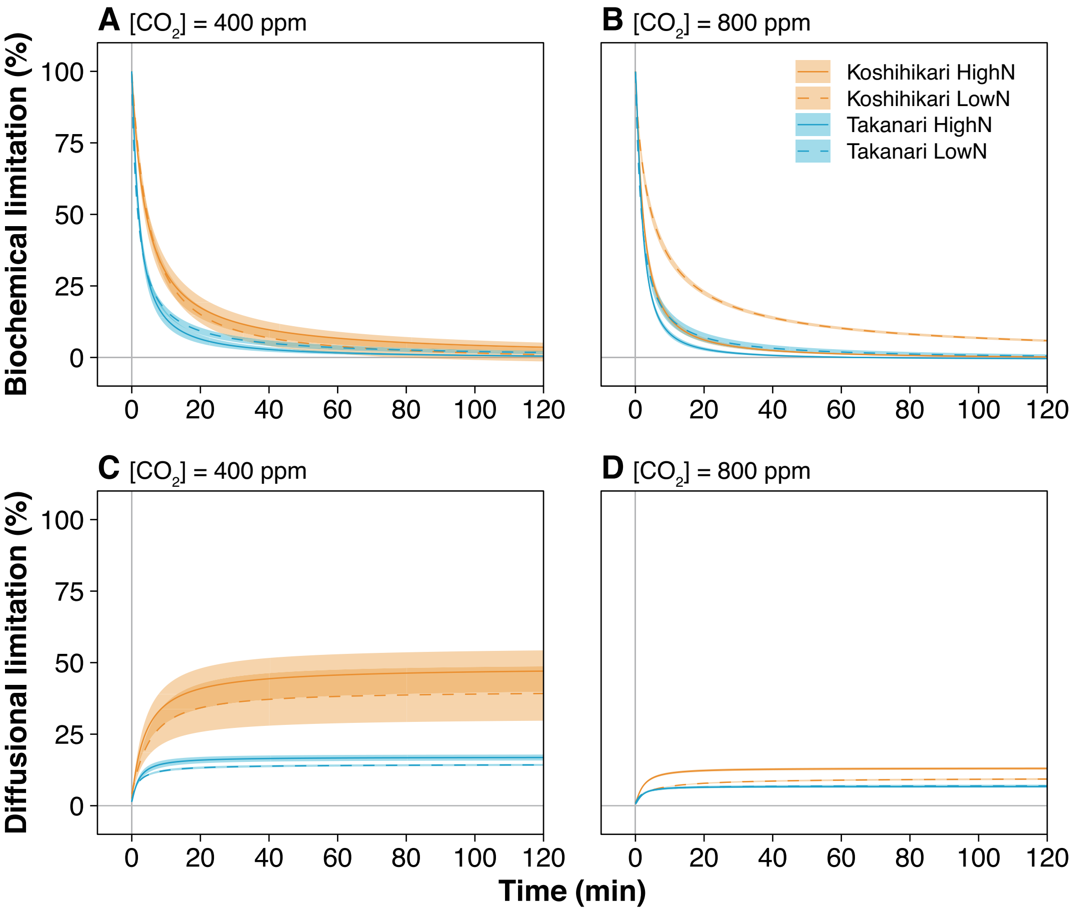


**Supplementary Figure S5** Biochemical limitation (**A**, **B**) and diffusional limitation (**C**, **D**) after a sudden increase in irradiance measured at different $CO_2$ concentrations. Means ± SE, $n = 4$.

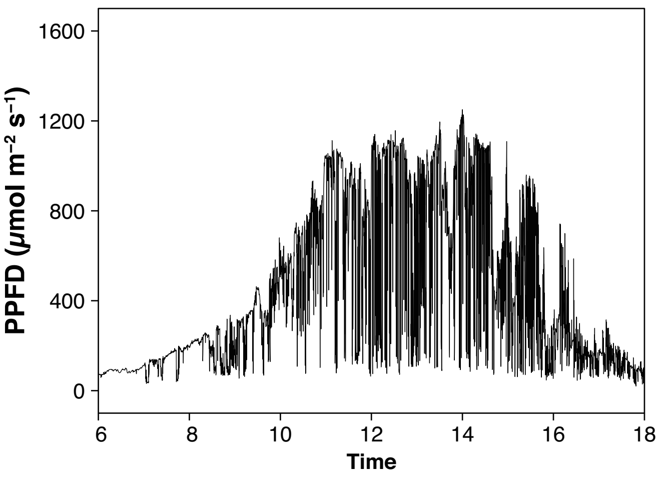


**Supplementary Figure S6** Diurnal pattern of photosynthetic photon flux density (PPFD) recorded at the top of the rice canopy.

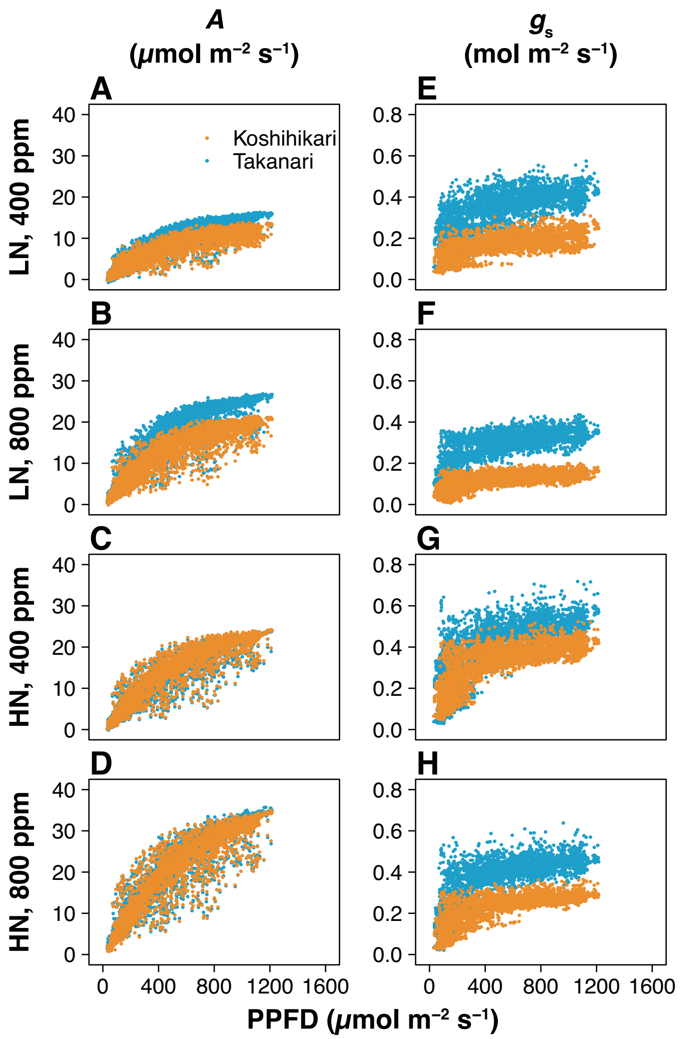


**Supplementary Figure S7** Relationship between gas exchange parameters and photosynthetic photon flux density (PPFD) of Koshihikari and Takanari under simulated lighting conditions in the LI6400 chamber. (**A–D**) $CO_2$ assimilation rate (*A*). (**E–H**) Stomatal conductance ($g_s$). The combination of the levels of N fertilization (LN, low N supply; HN, high N supply) and $CO_2$ concentrations (400 or 800 ppm) is shown on the left. Values are means ($n = 4$).

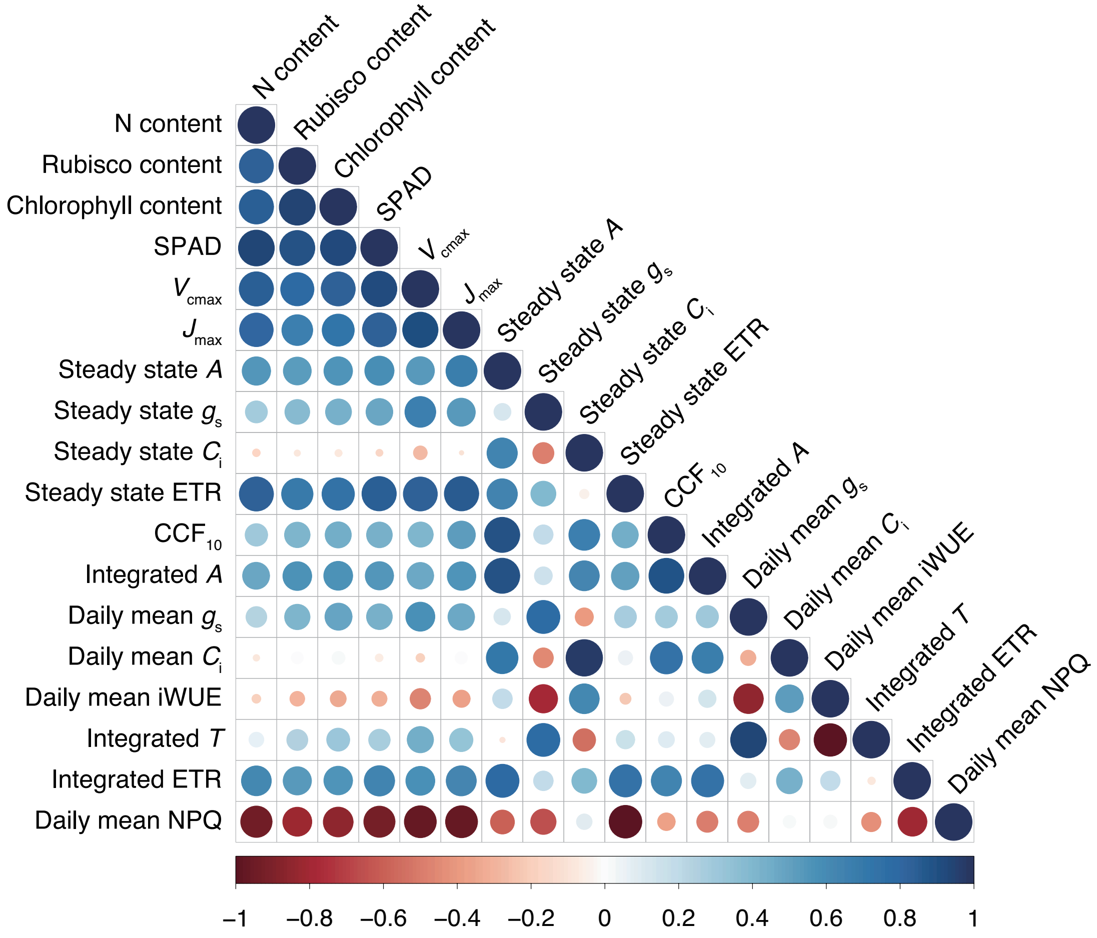


**Supplementary Figure S8** Pearson correlation coefficients of leaf contents of N, Rubisco, and chlorophyll and photosynthetic parameters measured in this study. $V_{cmax}$, maximum rate of carboxylation; $J_{max}$, maximum rate of electron transport; $A$, $CO_2$ assimilation rate; $g_s$, stomatal conductance; $C_i$, internal leaf $CO_2$ concentration; ETR, electron transport rate; $CCF_{10}$, cumulative $CO_2$ fixation during the first 10 min after transition from low to high irradiation; iWUE, intrinsic water use efficiency; $T$, transpiration rate; NPQ, nonphotochemical quenching. Daily mean values and integrated values are means and accumulated values under simulated light conditions in the LI6400 chamber.

**Supplementary Table 1** Daily means of stomatal conductance ($g_s$), internal leaf $CO_2$ concentration ($C_i$), intrinsic water use efficiency (iWUE), nonphotochemical quenching (NPQ) and integrated values of transpiration ($T$) and electron transport rate (ETR)

| Nitrogen | LN | | | | HN | | | |
|---|---|---|---|---|---|---|---|---|
| $CO_2$ | 400 ppm | | 400 ppm | | 400 ppm | | 400 ppm | |
| Cultivar | Koshihikari | Takanari | Koshihikari | Takanari | Koshihikari | Takanari | Koshihikari | Takanari |
| Daily mean $g_s$ (mol m$^{-2}$ s$^{-1}$) | 0.15 ± 0.00$^{ab}$ | 0.29 ± 0.04$^{cd}$ | 0.10 ± 0.01$^{a}$ | 0.25 ± 0.03$^{bcc}$ | 0.28 ± 0.03$^{bcc}$ | 0.37 ± 0.05$^{d}$ | 0.20 ± 0.02$^{ac}$ | 0.32 ± 0.03$^{cd}$ |
| Daily mean $C_i$ (ppm) | 322 ± 5$^{a}$ | 346 ± 2$^{a}$ | 605 ± 19$^{b}$ | 684 ± 11$^{c}$ | 325 ± 5$^{a}$ | 337 ± 3$^{a}$ | 648 ± 6$^{c}$ | 686 ± 4$^{c}$ |
| Daily mean iWUE ($\mu$mol mol$^{-1}$) | 42.1 ± 4.2$^{a}$ | 25.9 ± 1.4$^{a}$ | 115.9 ± 18.2$^{c}$ | 57.0 ± 6.4$^{ab}$ | 38.5 ± 1.8$^{a}$ | 30.2 ± 1.7$^{a}$ | 81.0 ± 3.8$^{b}$ | 55.4 ± 4.3$^{ab}$ |
| Integrated $T$ (mol m$^{-2}$ day$^{-1}$) | 98.9 ± 4.1$^{ac}$ | 149.5 ± 12.2$^{d}$ | 65.3 ± 8.1$^{a}$ | 130.5 ± 12.3$^{bcc}$ | 142.6 ± 9.6$^{cd}$ | 160.5 ± 11.7$^{d}$ | 88.1 ± 6.9$^{ab}$ | 142.1 ± 10.5$^{cd}$ |
| Integrated ETR (mol m$^{-2}$ day$^{-1}$) | 2.54 ± 0.12$^{a}$ | 2.80 ± 0.15$^{ab}$ | 3.17 ± 0.17$^{bc}$ | 3.43 ± 0.11$^{cd}$ | 3.65 ± 0.18$^{cd}$ | 3.19 ± 0.11$^{bc}$ | 3.90 ± 0.07$^{d}$ | 3.52 ± 0.06$^{cd}$ |
| Daily mean NPQ | 0.94 ± 0.05$^{b}$ | 0.86 ± 0.02$^{b}$ | 0.92 ± 0.05$^{b}$ | 0.88 ± 0.08$^{b}$ | 0.61 ± 0.06$^{a}$ | 0.75 ± 0.04$^{ab}$ | 0.73 ± 0.04$^{ab}$ | 0.79 ± 0.02$^{ab}$ |

Values are means ± SE ($n = 4$). Values followed by the same letter do not differ significantly among groups at $P < 0.05$ by Tukey–Kramer multiple comparison test.
